# The Spectrum of Acute Cerebrovascular Disease in Patients with COVID-19

**DOI:** 10.3390/biomedicines10020435

**Published:** 2022-02-13

**Authors:** Rachel Triay, Prabandh Buchhanolla, Alexas Gaudet, Victoria Winter, Alexandra Gaudet, Mehdi Faraji, Eduardo Gonzalez-Toledo, Harish Siddaiah, Hugo H. Cuellar-Saenz, Steven Bailey, Vijayakumar Javalkar, Rosario Maria S. Riel-Romero, Roger E. Kelley, Felicity N. E. Gavins, Junaid Ansari

**Affiliations:** 1 LSU Health Shreveport School of Medicine, Louisiana State University Shreveport, Shreveport, LA 71103, USA; ret002@lsuhs.edu (R.T.); akg002@lsuhs.edu (A.G.); vkw001@lsuhs.edu (V.W.); akg001@lsuhs.edu (A.G.); 2 Department of Neurology, Louisiana State University Health Sciences Center-Shreveport, Shreveport, LA 71103, USA; prabandh.buchhanolla@lsush.edu (P.B.); rosario.rielromero@lsuhs.edu (R.M.S.R.-R.); vijayakumar.javalkar@lsuhs.edu (V.J.); roger.kelley@lsuhs.edu (R.E.K.); 3 Department of Radiology, Louisiana State University Health Sciences Center-Shreveport, Shreveport, LA 71103, USA; mehdi.faraji@lsuhs.edu (M.F.); eduardo.gonzaleztoledo@lsuhs.edu (E.G.-T.); hugo.cuellarsaenz@lsuhs.edu (H.H.C.-S.); 4 Department of Anesthesiology, Louisiana State University Health Sciences Center-Shreveport, Shreveport, LA 71103, USA; harish.bangaloresiddaiah@lsuhs.edu; 5 Department of Cardiology, Louisiana State University Health Sciences Center-Shreveport, Shreveport, LA 71103, USA; steven.bailey@lsuhs.edu; 6 Centre for Inflammation Research and Translation Medicine, Department of Life Sciences, Brunel University London, Uxbridge SL0, UK; felicity.gavins@brunel.ac.uk

**Keywords:** COVID-19, cerebrovascular disease, acute ischemic stroke, seizures, stroke

## Abstract

(1) Background: COVID-19 infection is responsible for the ongoing pandemic and acute cerebrovascular disease (CVD) has been observed in COVID-19 patients. (2) Methods: We conducted a retrospective, observational study of hospitalized adult patients admitted to our hospital with SARS-CoV-2 and acute cerebrovascular disease. All clinical data were reviewed including epidemiology, clinical features, laboratory data, neuroradiological findings, hospital management and course from 32 patients hospitalized for COVID-19 management with acute cerebrovascular disease. (3) Results: Acute CVD with COVID-19 was associated with higher NIH stroke scale on discharge compared to non-COVID-19 CVDs. Seizures complicated the hospital course in 16% of COVID-19 patients with CVD. The majority of the acute CVDs were ischemic (81%) in nature followed by hemorrhagic (22%). Acute CVD with COVID-19 resulted in average hospital stays greater than twice that of the control group (13 days in COVID-19, 5 days in control). Acute CVD with COVID-19 patients had worse clinical outcomes with 31% patient deaths and 6% discharged to hospice. In the control group, 6% of patients died. (4) Conclusions: Acute CVD associated with COVID-19 tends to be more complicated with unique and adverse clinical phenotype, longer hospital admissions, and worse clinical outcomes.

## 1. Introduction

Coronavirus disease 2019 (COVID-19) infection caused by Severe Acute Respiratory Syndrome Coronavirus 2 (SARS-CoV-2) is responsible for the ongoing pandemic resulting in over 5 million deaths worldwide. Despite COVID-19 being an acute respiratory disease, it has a potential to cause a broad range of neurological manifestations due to neurotropism [1]. A wide range of neurological manifestations are known to be associated with COVID-19 infection, including, but not limited to, encephalopathy, seizures, cerebrovascular events, acute polyneuropathy, headache, and hypogeusia. The definitive mechanisms for COVID-19-related neuropathogenesis need further elucidation, including the differences between hematogenous versus transynaptic spread and the role of ACE2 receptors [2]. Specifically, acute cerebrovascular disease (CVD) has been observed in COVID-19 patients [3]. The incidence of new-onset CVD during COVID-19 infection has ranged from 0.5% up to 5.9% [4,5]. Acute CVD, particularly ischemic stroke, is more common in severe cases of COVID-19 infection [6]. Most of the cerebrovascular complications in the setting of COVID-19 are known to have significant unfavorable prognoses [3,6]. Notably, the increased risk of CVDs with COVID-19 infection can have many adverse outcomes resulting in increased morbidity and mortality compared with non-COVID-19 related CVDs [7].

In response to viral replication, COVID-19 infection causes a widespread pro-inflammatory and hypercoagulable state, leading to cytokine release and endothelial damage [8,9,10]. COVID-19 has been shown to cause venous and arterial thrombosis which is mediated by inflammation, endothelial dysfunction, thrombin generation, and platelet activation [11,12]. Therefore, due to such systemic thromboinflammatory responses, COVID-19 is known to cause severe cardiovascular disease and CVDs [13]. This can be observed through various changes in laboratory data indicating systemic inflammation and hypercoagulability, and studies of post-mortem histology showing macrovascular and microvascular thrombosis [14]. Interestingly, it has been shown that many patients present with acute CVD days before demonstrating symptoms of COVID-19, suggesting there may be an increased risk of thrombosis early in the disease [14]. One large New York study indicated that in COVID-19-related CVD, stroke was the principle reason for admission instead of respiratory symptoms in 44% of the cases [15]. Therefore, it is important to realize the presence of a stroke could be easily overlooked in severe COVID-19 patients who are intubated and sedated [15].

## 2. Materials and Methods

We conducted a single-center retrospective, observational study of hospitalized adult patients (age > 18 years) admitted to OLSU-S, Shreveport, LA, USA with laboratory-confirmed SARS-CoV-2 and acute cerebrovascular disease between 15 March 2020 to 15 September 2021. The study was approved by the institutional review board of the LSUHS (STUDY00001451) and conducted in accordance with the Declaration of Helsinki. A total of 777 patients fitting these criteria were admitted to OLSU-S with acute COVID-19 diagnosis during this period. All clinical data were reviewed including epidemiology, clinical features, laboratory data, neuroradiological findings, hospital management and course from 32 patients hospitalized for COVID-19 management with acute cerebrovascular disease at OLSU-S and 36 age- and sex-matched patients hospitalized during the same time period with non-COVID cerebrovascular disease. 

All the clinical information including demographic characteristics, medical history, clinical signs and symptoms, laboratory findings, radiological findings, medical management and clinical course was obtained from Epic Hyperspace electronic medical records system. 

Data are represented as number and percentage for categorical variables or mean and standard deviation for continuous variables. Comparisons of dichotomous variables between patients with and without COVID-19 were analyzed using the Chi square test. Continuous variables were analyzed using the independent sample T-test. Logistical regression was performed for adjusting age, sex, ethnicity and comorbidities. Level of significance was established at the 2-sided 5% level. Analyses were performed using SPSS v27.

## 3. Results

### 3.1. Etiology and Risk Factors of Acute CVD and COVID-19

Studies have been ongoing throughout the COVID-19 pandemic analyzing the incidence of acute CVD in COVID-19 patients [3]. The incidence of patients with acute CVD in COVID-19 patients admitted to OLSU-S was 4.1%.

In our acute CVD and COVID-19 cohort at OLSU-S, 22 (68.75%) of the patients were male and, 18 (56%) were Black or African American. The most common co-morbidities seen in our acute CVD and COVID-19 patients were 22 (69%) with hypertension, 12 (37.5%) with obesity (with an average BMI of 31 kg/m^2^), 10 (31%) with hyperlipidemia, 10 (31%) former or current cigarette smokers, nine (28%) with diabetes mellitus, nine (25%) with atrial fibrillation and five (16%) with a history of coronary artery disease. These are all known to be risk factors for acute CVD, but likely accelerated the risk in association with COVID-19 infection [16,17] (Table 1).

### 3.2. Clinical Manifestations and Prognosis of Patients with Acute CVD and COVID-19

In our acute CVD and COVID-19 infection cohort at OLSU-S, initial presentation tended to be severe, with average initial National Institute of Health stroke scale (NIHSS) of 11.7. The most common neurological symptoms were weakness (84%, 27 patients), dysarthria (62%, 20 patients), aphasia (53%, 17 patients), impaired consciousness (44%, 14 patients), vision impairment (16%, five patients), and dizziness (12%, four patients). Seizures complicated the hospital course of five (16%) of our patients. EEG findings ranged from mild to severe encephalopathy, with focal slowing in two patients. The severity of clinical courses is represented by nine (28%) of our patients experiencing encephalopathy and six (19%) experiencing delirium (Table 1).

**Figure 1:**
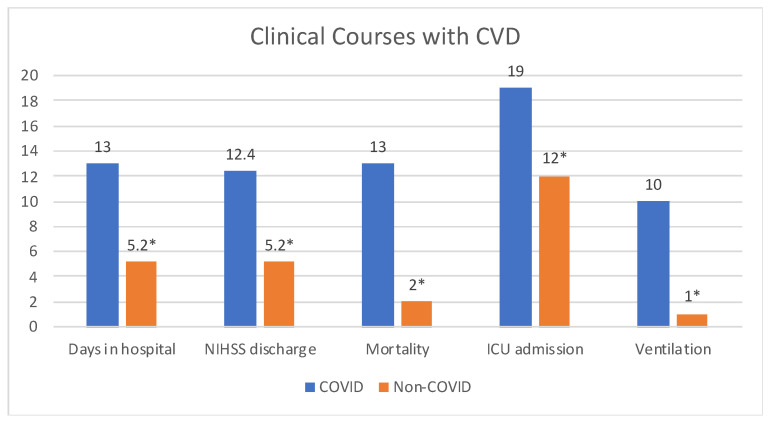
Clinical Courses of CVD patients with (*n* = 32) and without (*n* = 36) COVID-19 infection. Patients diagnosed with CVD during acute COVID-19 infection had longer hospital stays, worse NIHSS at discharge, greater mortality, increased ICU admissions and ventilation. * All data are significant with *p* < 0.05.

Our cohort was hospitalized for an average of 13 days (ranging from 1 to 67 days). Clinical outcomes were more complicated with greater mortality and morbidity in the COVID-19 patients with CVD compared to non-COVID-19 patients with CVD, as 19 (59%) required ICU admission, 10 (31%) required mechanical ventilation, and 13 (40%) patient died or were discharged to hospice care (OR = 11.6, 95% CI 2.3–57, *p* = 0.001) (Figure 1). Those patients who did recover and were discharged home tended to have high disability levels, as the average NIHSS_discharge_ was 12.4. NIH_d_ change in NIH stroke from admission to discharge was higher in COVID CVDs (+2.5) compared to non-COVID CVDs (−3.0). This was consistent with other studies having mortality rates ranging from 27.6% to 64% [12]. 19 (60%) patients with CVD and COVID recovered compared to 34 (94%) patients with non-COVID CVDs (Table 2). After adjusting for age, sex, race and comorbidities, odds for COVID patients with CVD is increased significantly in terms of length of stay (OR = 1.30, 95% CI 1.05–1.58, *p* = 0.01). Mortality (OR = 3.04, 95% CI 0.15–72, *p* = 0.41) and ICU admission (OR = 2.09, 95% CI 0.3–13, *p* = 0.42) also have increased odds but were not statistically significant.

### 3.3. Laboratory Data of Patients with Acute CVD and COVID-19

In our cohort of acute CVD and COVID-19 patients, laboratory trends were comparable to other studies. Systemic inflammatory markers tended to be elevated in the majority of our patients, with average maximum C-reactive protein (CRP) of 11.3 mg/dL, average maximum lactate dehydrogenase (LDH) of 460 U/L, and average maximum ferritin of 2287 ng/mL. The majority of our patients were hypercoagulable during admission and throughout their hospital stays, with average maximum D-dimer of 9792 ng/mL. Maximum average troponin in our patient cohort was 0.08 ng/mL, without any diagnosed co-morbid cardiac comorbidity. The majority of our patient cohort had normal white blood cell (WBC) counts, absolute neutrophil counts (ANCs), and absolute leukocyte counts (ALCs) on admission, showing no obvious immunosuppression initially. However, seven (21.8%) of our patients did present with an ALC less than 1 K/uL and many of our patients’ labs trended towards elevated WBCs and ANCs and decreased ALCs. Many of our patients also experienced acute kidney injury while hospitalized, with average maximum creatinine value of 2.3 mg/dL. Our cohort also had significant changes in procalcitonin levels, with average admission procalcitonin of 0.71 ng/mL and average maximum procalcitonin of 24.2 ng/mL (Table 3).

### 3.4. Neuroradiological Data of Patients with Acute CVD and COVID-19

**Figure 2:**
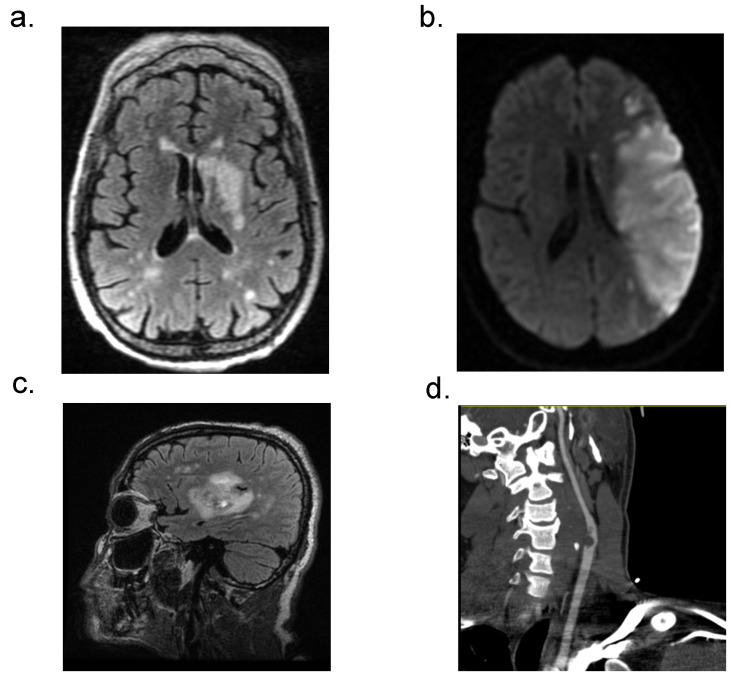
Neuroradiological manifestations of patients with CVD and acute COVID-19. (**a**) MRI FLAIR sequence with left basal ganglia and multifocal areas of ischemia. (**b**) MRI DWI image showing large left MCA territory infarct. (**c**) MRI FLAIR image of CVD in right basal ganglia (**d**) CTA image showing large clot in the internal carotid artery.

Our OLSU-S acute CVD and COVID-19 cohort consisted of unique and severe neuroradiological phenotypes (Figure 2). Acute ischemic strokes (AIS) were proven by neuroimaging in 26 (81.2%) of our patients. There was large vessel occlusion in 15 (46.8%) and large vessel stenosis in five (15.6%) of these patients. Of these, intravenous tissue plasminogen activator (TPA) was given in nine (28%) and mechanical thrombectomy completed in nine (28%). Carotid stents were required in two patients (6%). Severe and widespread ischemia was fairly common, with 12 (37.5%) cases consisting of multifocal ischemia. There were also seven patients (21.9%) with hemorrhagic transformation and seven (21.9%) with midline shifts. Interestingly, we had one patient with a free-floating thrombus in the internal carotid artery (ICA) requiring heparin infusion, which led to hemorrhagic transformation and midline shift. Additionally, we had one patient with a thalamic venous stroke who died, which is consistent with literature on high mortality in cases of cerebral venous thromboses. Our cohort also included two (6%) patients with intracranial hemorrhages, one which was an intraventricular hemorrhage and one with a basal ganglia hemorrhage.

## 4. Discussion

Using clinical information from the patients admitted to OLSU for acute CVDs and COVID-19 management, we have identified several key findings regarding CVD risk and acute COVID-19 management. Specifically, we found that (1) acute CVD associated with COVID-19 (compared with those without COVID-19) tends to be more complicated with adverse clinical phenotypes. (2) The majority of acute CVDs were ischemic in nature followed by hemorrhagic type. (3) Acute CVD in the setting of the COVID-19 group included unique neuroradiological phenotypes, including free-floating thrombi, intraventricular hemorrhage, thalamic venous stroke, and cases of multifocal ischemia. (4) Acute CVD with COVID-19 resulted in longer average hospital stays, which were greater than twice that of the control group. (5) Acute CVD with COVID-19 patients had worse clinical outcomes with more patient deaths and long-term hospice care compared to controls.

Initial studies have suggested an increase in incidence of acute CVDs with worse outcomes in the setting of COVID-19 infection [18,19,20]. Acute CVD with COVID-19 is more likely to be seen in patients who are older, male, have other cardiovascular risk factors and who have severe COVID-19 infection [15]. The most common cardiovascular risk factors were hypertension, obesity, diabetes mellitus, smoking history, and history of CVD, which are known to be associated with increased severity of COVID-19 [7]. Although there are reports of increased incidence of atrial fibrillation in patients with COVID-19, our study found no difference between patients with COVID-19 and controls [21]. Our study found acute CVDs in COVID-19 had a male patient predominance and increased incidence in Black or African American patients. The racial disparity in our institution is most likely related to our patient population, as our acute CVD without COVID-19 infection control group similarly consisted of majority Black or African American patients.

**Figure 3:**
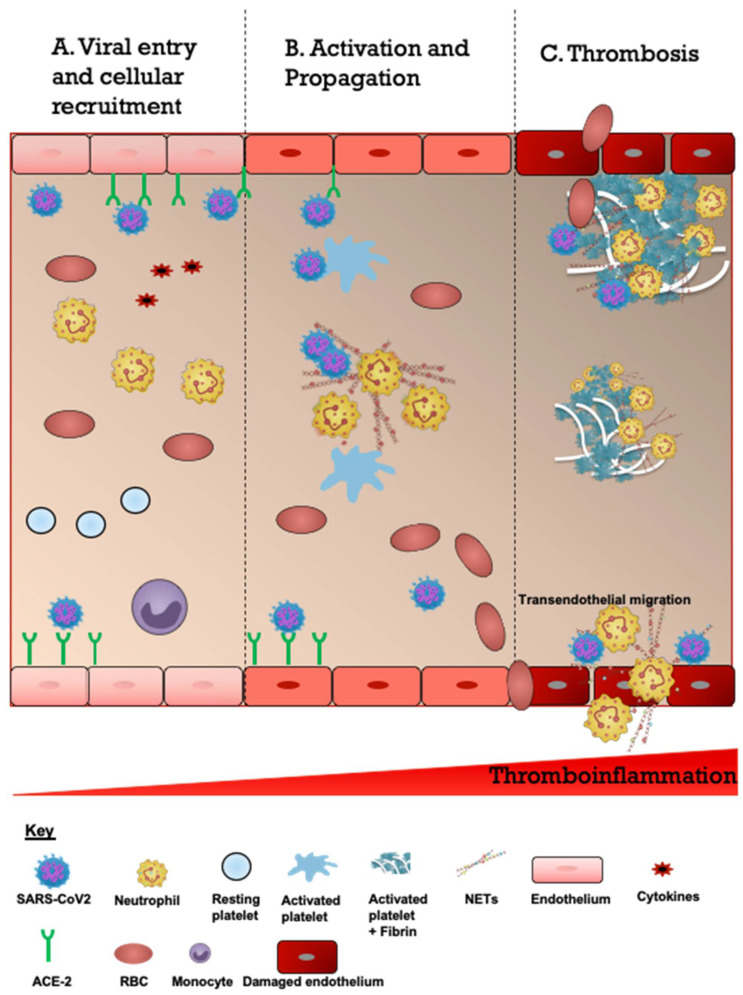
Schematic description of proposed overview of the COVID-19-associated CVD pathogenesis. (**A**). Viral entry and cellular recruitment. The entry of severe acute respiratory syndrome coronavirus 2 (SARS-CoV2) into the cerebrovascular system is followed by increased recruitment of neutrophils, platelets and erythrocytes. SARS-CoV2 binds to the ACE2 receptor expressed on the endothelial cells resulting in endothelial activation and cytokine release (cytokine storm). (**B**). Activation and propagation. Cytokines and SARS-CoV2 activate neutrophils to produce neutrophil extracellular traps which are laden with prothrombotic mediators such as neutrophil elastase, cathepsin G, histones (citH3), and coagulant factors (FV and FX). This also leads to activation of platelets. (**C**). Thrombosis. The subsequent responses consist of continuous accumulation of activated neutrophils, platelets and erythrocytes and engagement of coagulation cascade resulting in growing thrombi and fibrin scaffolds culminating in thrombosis and ischemic stroke. Some thrombi may detach and embolize to distal vessels. Endothelial cells are disrupted and damaged which increases the risk of intracerebral hemorrhage and transendothelial migration.

The ability of SARS-CoV2 to cause CVDs may be either directly via cellular transport or indirectly via infection of leukocytes especially neutrophils (Figure 3). SARS-CoV2 induces a systemic inflammatory and hypercoagulable response, conferring a high risk of thrombosis and cerebrovascular events [22]. Interestingly, there are studies which propose a mechanism linking cardiovascular risk factors to COVID-19’s affinity to the ACE2 receptor [14,15,23]. ACE2 expression was found to be increased in ischemic brains but was also upregulated in vessels of patients with diabetes and those exposed to cigarette smoke [14]. Based on this high expression of ACE2, smoking and diabetes may increase the ability of COVID-19 to enter and infect the brain [14]. These CVD risk factors, including smoking and diabetes, are likely associated with negative progression and adverse outcomes of COVID-19 [14]. One study proposed that patients with a history of CVD were 2.5 times more likely to develop severe COVID-19, which is also known to put patients at a higher risk for subsequent acute CVD [15].

Various observational studies have been reporting on the severity and clinical outcomes of acute CVD patients in the context of COVID-19 [3]. There is an overwhelming consensus that the overall clinical outcome is unfavorable with high mortality and morbidity rates, as well as large proportions of patients being discharged with severe disability [3]. Acute CVD is now known to worsen adverse outcomes in COVID-19 patients [24]. Stroke severity, which can be objectively measured by the NIHSS, is higher in patients with COVID-19, with studies reporting an average NIHSS of 10 to 17 [7,25]. Stroke symptoms have included sensory and motor deficits, with dysarthria being a commonly reported finding, but this can vary greatly due to the severe, widespread, and multifocal nature of COVID-19-related CVDs [13]. Patients with cerebral ischemia and COVID-19 have rapid clinical progression and increased thrombus burden, which results in worse clinical prognosis [24]. Our study revealed severe acute CVD presentation with higher initial NIHSS compared to controls. The common neurological symptoms were motor weakness followed by dysarthria, aphasia, impaired consciousness, vision impairment, and dizziness. The most common EEG findings ranged from mild to severe encephalopathy, followed by focal slowing. Our cohort of patients experiencing encephalopathy with delirium could be associated with the longer hospital stays required for acute CVD and COVID-19 patients.

Explanations for such poor prognoses include high incidence of emergent large vessel occlusion (LVO) and patients with acute infarcts in multiple vascular territories [24]. Massive cytokine release and thrombogenesis may contribute to this, as this stimulates accumulation of coagulation factors in plasma and on the surface of endothelial cells, inducing platelet aggregation in the endothelium [24]. Proposed explanations for the relation between COVID-19-associated ischemic strokes and increased stroke severity include direct vasculopathic effects, immune-mediated platelet activation, dehydration, and infection-induced cardiac arrhythmias [7]. Increased severity could also be attributed to social distancing and isolation, or hesitancy of going to a hospital, which may prevent patients from seeking care when they have smaller deficits [25]. There are also broad multi-system complications of COVID-19 including ARDS, arrhythmias, acute cardiac injury, pulmonary embolism, cytokine release syndrome, and secondary infection, which all contribute to mortality and morbidity [13].

The inflammatory and hypercoagulable state induced by COVID-19 led to an array of serum marker changes [11,15]. Significant markers which were commonly reported to be elevated in many studies of acute CVD and COVID-19 included ferritin, lactate dehydrogenase (LDH), D-dimer, C reactive protein (CRP), and troponin [11,15]. Patients with acute CVD have increased inflammatory responses, including higher white blood cell (WBC) count, absolute neutrophil counts (ANC), and CRP levels, but lower absolute lymphocyte counts (ALC), suggesting dysregulated immunity [15,26]. The cytokine storm associated with COVID-19 infection could also be responsible for elevations in CRP, which has been associated with increased risk of CVD and myocardial infarction even in healthy people [27]. Elevated D-dimer levels were suggestive of hypercoagulability, which was found to be significantly higher in patients experiencing acute CVD. It has been shown in multiple studies that D-dimer levels are significantly higher in COVID-19 patients with poor prognosis [28]. Additionally, acute CVD patients often have some degree of kidney injury, with elevated creatinine levels, and prolonged hospital courses [29]. Procalcitonin, another marker representing inflammation, is known to be associated with more severe cases of COVID-19, with more critically ill patients, and fatal outcomes [30,31].

Unique trends were noted on neuroradiology of COVID-19 patients. Occlusions occurred in multiple vascular territories. CVD was the primary neurological manifestation of COVID-19 in many patients [23]. Typical imaging findings of acute ischemic stroke (AIS) have been appreciated on non-contrast CT of the head (CTH) in COVID-19 patients, such as abnormal hypoattenuation of the brain parenchyma, loss of grey–white differentiation, and sulcal effacement [28]. However, CT angiography of some of these COVID-19 patients revealed extensive vascular occlusion disease. There have been many reports of large vessel occlusions (LVO), but also findings of markedly reduced flow even in the absence of LVO. There is evidence that thrombosis of extracranial carotid arteries may play a role in a subset of COVID-19 patients [28]. Cryptogenic stroke occurred twice as frequently in COVID-19-positive patients compared to COVID-19-negative patients. It was proposed that COVID-19-associated cryptogenic stroke represented a unique stroke mechanism associated with higher probability of early mortality [10]. Another reported cerebrovascular manifestation of COVID-19 is cerebral venous thrombosis, which has been associated with coagulopathy and multisystem organ failure [28]. Thromboses have been noted in dural venous sinuses, deep cerebral veins, and cortical veins. Notably, there has been a high mortality rate with these patients, possibly related to involvement of the deep venous system or non-neurologic complications of COVID-19 [28]. Other neuroradiologic findings reported include thrombotic microangiopathy, acute hemorrhagic necrotizing encephalopathy, hemorrhagic posterior reversible encephalopathy syndrome, diffuse leukoencephalopathy, and acute disseminated encephalomyelitis [28].

Due to the relatively short duration of the COVID-19 pandemic, therapeutics for acute CVD in the setting of acute COVID-19 are still under investigation. There are many different factors that must be considered when treating such patients, including the complicated phenotype of COVID-19-associated CVDs and other comorbid symptoms stemming from COVID-19 itself. However, these complicating factors should not defer or substantially change the management directed at the CVD [32]. Stroke care must be treated as an emergency in COVID-19 patients, just as it would be treated in non-COVID-19 patients. Patients with suspected or confirmed COVID-19 presenting with any signs or symptoms of acute CVD should receive standard of care imaging and be evaluated for intravenous thrombolysis and/or intra-arterial mechanical thrombectomy. It is also important for staff in isolation areas to be trained in early recognition and reporting of acute CVD, as COVID-19 increases risk of developing an acute CVD. There should be a low threshold for neurological consultation [32].

Some hospitals have reported increased door-to-needle times to accommodate for infection control measures and balance avoidance of risk of infectious exposure to stroke team members while attempting to reduce time to reperfusion [32]. The same pattern of delays in door-to-mechanical-thrombectomy time can be attributed to infection control measures [32]. However, overall, large studies show that there is similar door-to-CT, door-to-needle, and door-to-endovascular therapy times in the pre-COVID-19 era compared with the COVID-19 era. Additionally, there were no significant differences in the proportion of patients who received intravenous thrombolysis or endovascular therapy [33].

Mechanical thrombectomy should be considered for patients, especially younger population who are possible candidates despite a COVID-19 diagnosis [32]. However, the efficacy of mechanical thrombectomy may be decreased in COVID-19 patients due to the tendency for multiple arterial occlusions, high clot burden, and clot fragmentation. Since COVID-19 increases the risk of thromboembolism, it is thought that thrombolysis is effective despite the underlying inflammatory component of COVID-19, which is routinely associated with unfavorable outcomes. In general, neurocritical care should continue to be provided to COVID-19 and acute CVD patients who may benefit from intensive care, with added infection control measures. Primary and secondary stroke prevention guidelines should be implemented, and early rehabilitation should be started in the isolation area if feasible [32]. There have been reports of decreases in prescription of antithrombotics at discharge, dysphagia screening, smoking cessation counseling, stroke education, and rehabilitation consideration in COVID-19 and acute CVD patients [33]. It is important to remember the established practices in treating acute CVD, even in a complicated COVID-19 patient. While traditional CVD treatments are still in place for patients with COVID-19, it is still unclear what is best, consequently ongoing research must continue [33].

## 5. Limitations

This study has various limitations. Only 32 acute CVD with COVID-19 patients and 36 acute non-COVID-19 controls were studied. Although patients in our hospital presented with COVID-19 and acute neurological deficits, the direct association of the SARS-CoV2 and neurological outcomes need more prospective and larger studies. Most of the clinical information was obtained from the electronic medical record system and therefore there is a risk of missing clinical features which might not have been obtained during clinical examination. Advanced neuroimaging such as MRI was not obtained in some patients due to risk of exposure and cross-infection especially in patients where it would not have changed clinical outcome or due to body habitus which was incompatible with the hospital’s MRI machine. Long-term follow-up and natural history have not been acquired. Lastly, it is important to know our study is based on the earlier variants of SARS-CoV2 and may not reflect the clinical phenotype of the current dominant omicron variant.

## 6. Conclusions

In conclusion, acute CVD with COVID-19 appears to be more complicated and severe, resulting in an adverse clinical phenotype, longer hospital admissions, and worse clinical outcomes. These patients may need more acute and frequent monitoring to avoid delayed management. Acute CVD in the setting of COVID-19 should be considered an important negative prognostic factor. Our study offers more evidence about the severity of cerebrovascular disease in COVID-19 infection and should help healthcare providers to decide on clinical management. 

## Figures and Tables

**Table 1:** Demographic and clinical features of patients with CVD with and without COVID-19.

	COVID (*n* = 32)	Non-COVID (*n* = 36)	*p* Value (Chi Square)
Age	63	64	0.68
Sex			
Female	10 (32%)	15 (42%)	0.37
Male	22 (68%)	21 (58%)	0.37
Race			
African American/Black	18 (56%)	26 (72%)	0.68
Caucasian/White	6 (24%)	9 (25%)	0.68
Other	2 (8%)	1 (3%)	0.68
Comorbidities			
Hypertension	22 (69%)	33 (92%)	<0.001
Hyperlipidemia	10 (33%)	18 (50%)	<0.001
Diabetes	9 (28%)	12(33%)	<0.001
Atrial fibrillation	8 (25%)	9 (25%)	1.000
Coronary artery disease	5 (16%)	7 (19%)	<0.001
Smoking	10 (31%)	17 (47%)	0.07
Obesity	12 (37%)	14 (38.9)	0.05
Chronic kidney disease	4 (13%)	3(8.3%)	<0.001
Malignancy	3 (9%)	2 (6%)	<0.001
Clinical Features			
Weakness	27 (84%)	30 (83%)	0.02
Impaired Consciousness	14 (44%)	5 (14%)	0.001
Dysarthria	20 (62%)	23 (64%)	0.13
Aphasia	17 (53%)	15 (42%)	0.05
Vision Impairment	5 (16%)	6 (17%)	0.05
Dizziness	4 (12%)	3 (8.3%)	0.004
Delirium	6 (19%)	0 (0%)	0.001
Seizures	5 (16%)	1 (3%)	0.02

**Table 2:** Descriptive statistics of clinical outcomes of patients with CVD with and without COVID-19.

	COVID (*n* = 32)	Non-COVID (*n* = 36)	*p* Value
NIH_initial_ (mean)	11.7	8.2	0.08
NIH_discharge_ (mean)	12.4	5.2	0.004
NIH_d_ (mean)	2.5	−3.0	0.01
Days in Hospital (mean)	13	5.2	0.002
ICU admission	19 (59%)	12 (33%)	0.04
Ventilation	10 (31.3%)	1 (3%)	<0.001
Outcomes			
Recovered	19 (60%)	34 (94%)	0.001
Death	13 (40%)	2 (6%)	0.001

NIH_initial_: Initial NIH stroke scale; NIH_discharge_: NIH stroke scale on discharge; NIH_d_: Change in NIH stroke scale from admission to discharge.

**Table 3:** Laboratory features of patients with CVD with and without COVID-19.

	COVID (*n* = 32)	Non-COVID (*n* = 36)	*p* Value (*t*-Test)
WBC	8.40 (±4.2)	8.1 (±2.71)	0.78
ANC	6.50 (±4.7	5.3 (±2.6)	0.22
ALC	1.30 (±0.5)	1.9 (±0.9)	0.003
NLR	6.67 (±4.4)	3.7 (±3.3)	0.02
Platelets	260.5 (±106.2)	258 (±78)	0.91
D-Dimer	9792 (±19k)	NR	
CRP	11.3 (±8.7)	NR	
Serum Creatinine	2.3 (±2.3)	1.1 (±0.5)	0.006
LDH	460 (±232)	NR	
Ferritin	2287 (±5244)	NR	
PT	13.6 (±2.4)	15 (±12)	0.05
INR	1.20 (±0.21)	1 (0.1)	0.07
APTT	42 (±33)	32 (±15)	0.15
Troponin_Max_	0.08 (±0.16)	0.02(±0.75)	0.58

WBC: white blood count; ANC: absolute neutrophil count, ALC: absolute lymphocyte count, NLR: Neutrophil lymphocyte ratio; CRP: C-reactive protein, LDH: lactate dehydrogenase, PT: Prothrombin time, INR: International normalized ratio, APTT: activated partial thromboplastin time, NR: not reported.

## Data Availability

Data can be provided on request.
